# Metabolic plasticity and adaptive evolution in *Fragaria vesca*: bridging wild diversity to crop improvement

**DOI:** 10.3389/fpls.2025.1729002

**Published:** 2026-01-21

**Authors:** José E. Pérez-Martín, Dries Bonte, Sonia Osorio, David Posé

**Affiliations:** 1 Instituto de Hortofruticultura Subtropical y Mediterránea “La Mayora”. Departamento de Biología Molecular y Bioquímica, Universidad de Málaga-Consejo Superior de Investigaciones Científicas (IHSM-UMA-CSIC), Málaga, Spain; 2 Department of Biology, Terrestrial Ecology Unit, Ghent University, Gent, Belgium

**Keywords:** environmental adaptation, fragaria vesca, genotype-by-environment, GWAS, local adaptation, metabolomics, phenotypic plasticity, rosaceae

## Abstract

Plants resilience capacity to environmental variability involves both phenotypic plasticity and genetic differentiation through evolutionary selection. Metabolism, particularly primary metabolism, plays a central role in the plant-environment interface, translating environmental and genetic signals into physiological, developmental, morphological and life history responses. The woodland strawberry, *Fragaria vesca*, serves as an ideal model system for studying these integrated processes due to its wide ecological distribution, high natural variation, sequenced diploid genome, and experimental tractability. This has resulted in a growing interest in integrating approaches from ecophysiology, metabolomics, and genomics to dissect these complex interactions. This review presents current knowledge on how adaptive phenotypic plasticity and genetic variation contribute to environmental adaptation in plants, with a particular focus on *F. ves*ca, discussing insights from studies examining plasticity along environmental gradients and from metabolomic genome-wide association studies (mGWAS), which integrate metabolomic profiling with GWAS to identify genetic loci underlying metabolite variation and metabolic pathway diversity. This integrative understanding provides a roadmap for leveraging wild genetic resources to enhance crop resilience in changing environments.

## 1 Introduction

Environmental heterogeneity imposes divergent selective pressures on natural populations. As a result, evolutionary adaptation, the process whereby populations evolve genetically based traits that confer higher fitness in their specific habitats, is often crucial for the long-term persistence and resilience of species (Hereford, 2009; Fournier-Level et al., 2011; Wadgymar et al., 2017). Several studies, particularly within the plant kingdom, demonstrate that populations perform best in their native conditions, highlighting the prevalence and significance of local adaptation (Leimu and Fischer, 2008). This adaptation is especially important when organisms need to adapt to novel conditions, but may fail when the pace of environmental changes exceeds the potential of adaptation (Jump and Peñuelas, 2005; Anderson and Song, 2020).

Phenotypic plasticity, which is the capability of a single genotype to exhibit a range of phenotypes in response to different conditions, is a key mechanism to cope with environmental change (Bradshaw, 1965; Pigliucci, 2001; Nicotra et al., 2010). For sessile organisms such as plants, the inability to actively relocate as individuals accentuates the importance of mechanisms that allow *in situ* response, such as plasticity and evolutionary adaptation (Huey et al., 2002). Their survival and reproductive success are directly driven by their capacity to cope with the existing local conditions through either immediate physiological adjustments and/or longer-term evolutionary changes (Schlichting, 1986; Sultan, 2000; Savolainen et al., 2013; Miryeganeh, 2021).

On the one hand, evolutionary adaptation, which involves changes in allele frequencies over generations, leads to genetically distinct populations specialized for their respective local environments and is underpinned by genotype-by-environment interactions (Anderson et al., 2011; Hoban et al., 2016; Yeaman, 2022). On the other hand, phenotypic plasticity allows organisms to develop rapid, reversible adjustments, often crucial for their persistence and opening the way for further adaptive responses (Pigliucci, 2005; Aubin-Horth and Renn, 2009; Nicotra and Davidson, 2010; Donnelly et al., 2012; Scheiner, 2016).

Historically, genetic differentiation has been considered as the principal driver of adaptive evolution. However, in the past few decades, a paradigm shift occurred, recognizing the main role of phenotypic plasticity not only as an alternative but also as a facilitator of evolutionary diversification (Pigliucci et al., 2006). Plasticity can help populations to cope with environmental change, maintain fitness in novel conditions, and even guide the direction of subsequent genetic adaptation, revealing its dual role in both immediate stress protection and long-term evolutionary driving (Price et al., 2003; Pfennig et al., 2010; Gomez-Mestre and Jovani, 2013).

A main concept underlying both plastic and genetically based adaptation is phenotypic matching between organism and environment, whereby performance is maximized when phenotypes align with environmental conditions. If we talk about phenotypic traits and delve into the core of them, we find metabolism, which constitutes itself a phenotypic group that directly influences other complex traits under selection. Metabolism is responsible for translation of genetic information and integration of environmental inputs into the organism’s biochemical and physiological profile, directly influencing species fitness (Fiehn, 2002; Hochachka and Somero, 2002; Brown et al., 2004; Sweetlove et al., 2008; Kooke and Keurentjes, 2012; Fernie and Tohge, 2017). Many adaptive responses, particularly plastic ones, involve dynamic metabolic reconfiguration: altered carbon partitioning, changes in photosynthetic capacity, or the induced production of defensive compounds (Ackerly et al., 2000; Caretto et al., 2015). These responses can be either immediate and reversible, delayed but sustained, or genetically fixed, depending on both the environmental regime and genetic background. Then, a mechanistic understanding of adaptation requires elucidating how environmental cues modulate metabolic pathways and how this modulation is shaped by genetic variation (Soltis and Kliebenstein, 2015).

## 2 Phenotypic plasticity: the flexible response to the environment

**Figure 1:**
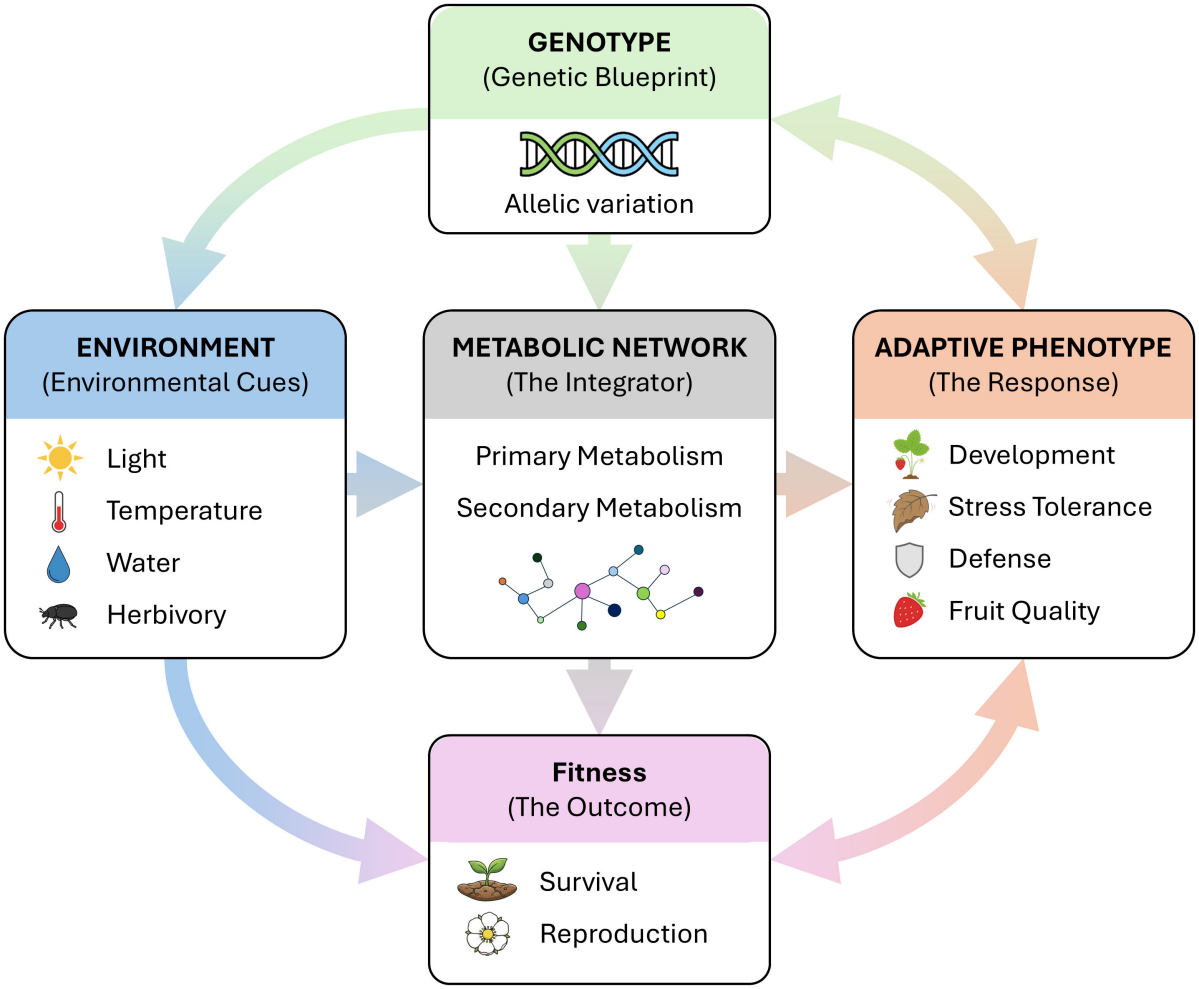
Conceptual framework illustrating how genotype-by-environment interactions through metabolic networks. Environmental cues activate signal transduction cascades that modulate metabolic pathways via post-translational, transcriptional, and epigenetic mechanisms. Genetic variation determines the capacity for metabolic plasticity, with different genotypes exhibiting distinct reaction norms along environmental gradients. The integration of primary and secondary metabolism translates these responses into adaptive phenotypes affecting growth, stress tolerance, defense capacity, and fruit quality. Phenotype-environment alignment ultimately determines fitness. Bidirectional arrows indicate evolutionary feedback through selection and plant-environment feedbacks.

As previously mentioned, phenotypic plasticity is crucial for adaptation to environmental changes and organisms’ resilience. **Figure 1** presents a conceptual framework illustrating how environmental cues and genetic background interact through metabolic networks to shape adaptive phenotypes and plant fitness.

### 2.1 Fundamental concepts and evidence of plasticity in plants

Phenotypic plasticity often serves as an initial adaptive strategy for populations to cope with environmental stress, providing the time needed for slower genetic adjustments to occur across generations (Yeh and Price, 2004; Ghalambor et al., 2007; Ho and Zhang, 2018). These plastic responses can be classified based on their fitness consequences. Adaptive plasticity occurs when the environmentally induced phenotypic change enhances the organism’s fitness in that specific environment (Sultan, 2000; Ghalambor et al., 2007), whereas maladaptive plasticity describes responses that reduce fitness, which can occur when environmental cues are unreliable (Ghalambor et al., 2007).

Several stresses trigger plastic responses, modulating different aspects of the plant physiology. In response to light availability, plants adjust leaf morphology, specific leaf area (SLA), chlorophyll content, and stem elongation to optimize light capture and prevent photodamage (Givnish, 1988; Sultan and Bazzaz, 1993a; Smith, 2000). Under drought, a wide array of responses is triggered, including stomatal regulation to control water loss, osmotic adjustments to maintain cell turgor, and changes in root morphology to enhance water acquisition (Sultan and Bazzaz, 1993b; Chaves et al., 2003; Nicotra and Davidson, 2010). Plants also exhibit remarkable plasticity in root growth and architecture under heterogeneous nutrient availability, concentrating root proliferation in nutrient-rich patches to enable more efficient foraging (Drew, 1975; Hutchings and de Kroon, 1994; López-Bucio et al., 2003). Temperature fluctuations trigger the synthesis of heat shock proteins and cold hardening processes (Thomashow, 1999; Sung et al., 2001), as well as phenological shifts that align reproduction with favorable conditions (Rathcke and Lacey, 1985; Franks et al., 2007).

Finally, biotic pressures such as herbivory or pathogen attack elicit a suite of plastic responses, including chemical and physical defenses that deter attacks and enhance resilience (Agrawal, 1999; Karban and Baldwin, 2007; War et al., 2012).

However, phenotypic plasticity is not without limitations. Its evolution is constrained by physiological costs and trade-offs, as maintaining the underlying mechanisms demands energy and resources. Consequently, some traits exhibit limited plasticity due developmental or structural constraints (DeWitt et al., 1998; Auld et al., 2010; Murren et al., 2015).

### 2.2 The study of plasticity: disentangling genetic and environmental effects

Investigating phenotypic plasticity requires carefully designed experiments that partition phenotypic variance into its genetic and environmental components, finding three main different alternatives.

**Figure 2:**
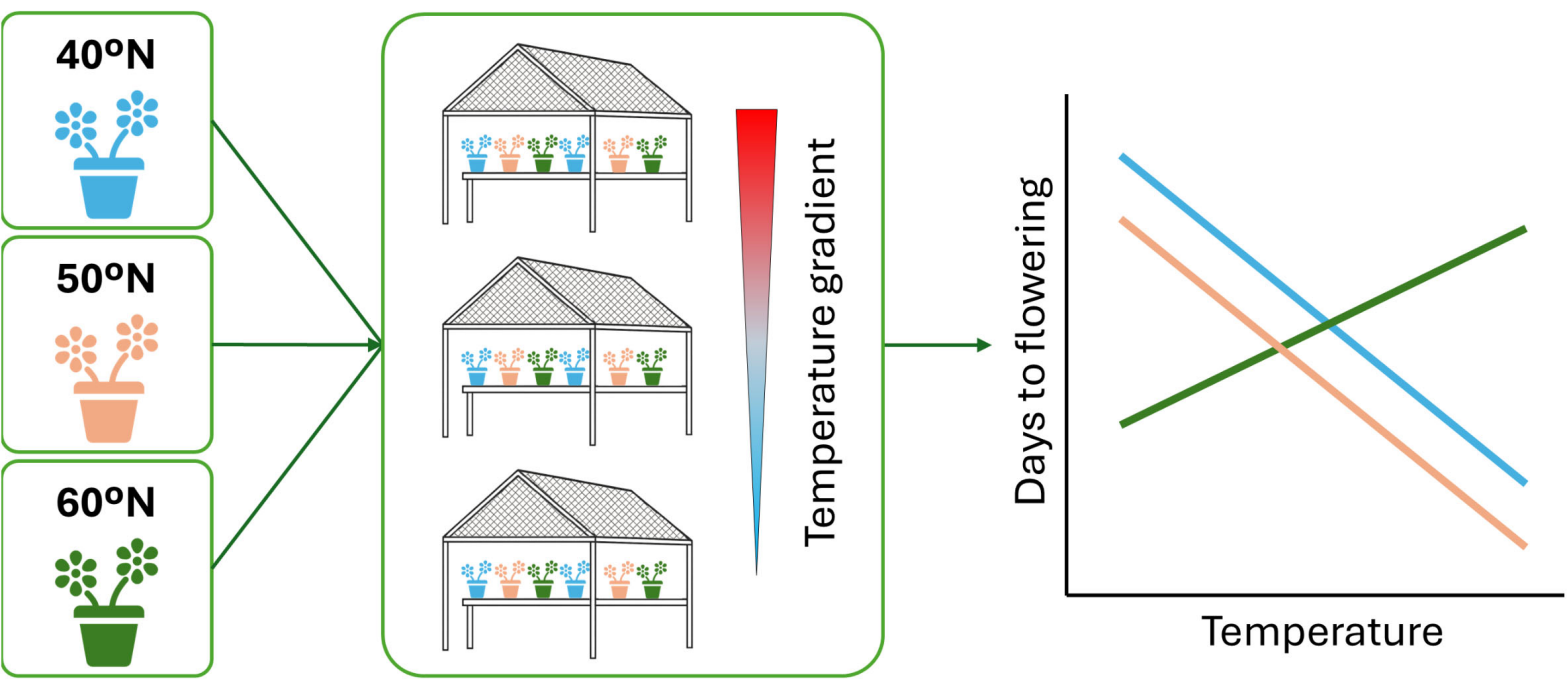
Schematic representation of a common garden experiment. Three populations from different latitudes [blue (40°N), orange (50°N), green (60°N)] are grown across three common garden locations (G1, G2, G3) established along a temperature gradient from warm (red, G1) to cold (blue, G3). The resulting reaction norms illustrate divergent phenotypic responses (e.g., days to flowering) across environments. Crossing reaction norm slopes demonstrate genotype-by-environment interaction (G×E): genotypes differ in their sensitivity to temperature, and relative ranking changes across environments.

- Common Garden Experiments: Classical approach, pioneered by (Clausen et al., 1940). Individuals from different populations (or genotypes) are grown together in one or more controlled or semi-controlled environments. When all individuals are grown under the same environmental conditions, any consistent phenotypic differences observed among populations (or genotypes) can be attributed to genetic differentiation. Establishing multiple common gardens across a range of environments allows to plot the phenotypic trajectory of each genotype as a ‘reaction norm’, which is a graphical representation of the range of phenotypes a single genotype expresses across different environments (Sarkar and Fuller, 2003). The slope and shape of this norm of reaction quantify the degree and nature of plasticity for a specific trait (**Figure 2**). Importantly, these designs must also account for maternal environmental effects, which may influence offspring phenotypes independently of genotypes and lead to misinterpretations if they are not controlled.
- Reciprocal Transplant Experiments: This approach extends the common garden design by planting individuals from different populations into each other’s native habitats, as well as back into their own as controls (Kawecki and Ebert, 2004; Hereford, 2009). Reciprocal transplants are particularly powerful to assess local adaptation, as it directly tests whether “local” genotypes outperform “foreign” ones in their home environments. They also provide an opportunity to observe plastic responses under the full complexity of natural environmental variation. While this method is logistically more challenging, it allows testing if environment-phenotype matching is actually driven by phenotypic plasticity, or in contrast, it is due to genotypic structure acquired among historic selective pressure, offering strong inference power regarding the adaptive significance of both genetic differentiation and phenotypic plasticity under ecologically realistic conditions.
- Controlled Environment Studies: Laboratory or growth chamber experiments enable precise manipulation of individual environmental factors while maintaining other constant (Kramer, 1978; Koch et al., 1987). This controlled setting allows researchers to isolate the effect of single environmental variables on trait expression and allows detailed characterization of genotype-specific reaction norms for those factors.

These experimental approaches, often complemented by quantitative genetic analyses, facilitate the statistical partitioning of observed phenotypic variance (P) into its components: genetic variance (G), environmental variance (E), and genotype-by-environment interaction variance (G×E). This partitioning accentuates the fact that plasticity arises from environmental effects on phenotype, while divergent reaction norms reveal unique behaviors of individuals, opening the way for natural selection to act on plastic responses.

### 2.3 Genotype-by-environment interaction (G×E): the evolutionary substrate for plasticity

G×E describes how different genotypes respond differently to the same environmental gradient, being graphically evidenced by divergent slopes or intercepts of reaction norms (Via and Lande, 1985; Hill and Mackay, 2004; Manuck, 2010; Nicotra et al., 2010). As G×E reveals genetic variation in the shape and magnitude of reaction norms, plasticity is therefore heritable and can evolve through natural selection maximizing fitness across a population’s environmental range (Via and Lande, 1985; Gomulkiewicz and Kirkpatrick, 1992; Scheiner, 1993). When reaction norms for fitness intersect, G×E indicates local adaptation: genotypes perform best in their native habitats but worse elsewhere, reflecting genetically encoded differences in optimal phenotypes rather than plastic adjustments (Hereford, 2009). Moreover, by favoring different genotypes under varying spatial or temporal conditions, G×E promotes the maintenance of genetic diversity within populations, preventing any single genotype from universally dominating (Ellner and Hairston, 1994; Gillespie and Turelli, 1989). This diversity of reaction norms also modulates population resilience: genotypes with broad, adaptive plasticity, or a mosaic of reaction shapes, are better equipped to cope with novel or shifting environments, such as those imposed by climate change (Sultan and Bazzaz, 1993a, 1993b; Reed et al., 2011; Lázaro-Nogal et al., 2015). Understanding G×E can then be applied in plant breeding, developing conservation strategies focused on preserving populations with high adaptive potential (Basford and Cooper, 1998; De Leon et al., 2016; Xu et al., 2017; Cooper and Messina, 2021; Yadesa, 2022).

Summarizing, far from being a mere statistical abstraction, G×E demonstrates that phenotype emerges through the interplay of genetic and environmental influences, and that the sensitivity of a genotype to its environment is itself genetically encoded. Recognizing G×E for adaptive traits reveals the genetic architecture of environmentally responsive phenotypes and opens the way for molecular investigations into the specific genes, regulatory networks, and metabolic pathways underlying plasticity.

## 3 *Fragaria vesca*: an emerging model system for eco-genomics

To explore the complex interplay between genotype, environment, and adaptive phenotypes, a tractable model with enough ecological and genetic variation is required. In this context, the woodland strawberry, *Fragaria vesca*, emerges as an ideal eco-genomic platform.

### 3.1 Species profile

*F. vesca* is a low‐growing, herbaceous perennial plant of the Rosaceae family, easily recognized by its trifoliate serrated leaves. Its flowering period is comprised during temperate seasons, occurring mainly in spring, but some ecotypes like *F. vesca* f. *semperflorens* flower continuously (Slovin et al., 2009). Following fertilization, flowers expand into the red accessory fruit (aggregate of achenes) renowned for its aroma and flavor (Yadav et al., 2019; Liu et al., 2020b).

Relevant to its employment as a research model, *F. vesca* presents a dual reproductive strategy, which can be (i) sexual reproduction via seeds, and (ii) clonal propagation through stolons, commonly known as runners (Staudt, 1999; Schulze et al., 2012). This clonal capacity facilitates rapid colonization of suitable microhabitats, significantly contributing to the species’ local persistence and spatial dynamics, often leading to the formation of extensive patches of interconnected individuals (Roiloa and Retuerto, 2006; Schulze et al., 2012).

### 3.2 Ecological amplitude and intraspecific variability

**Figure 3:**
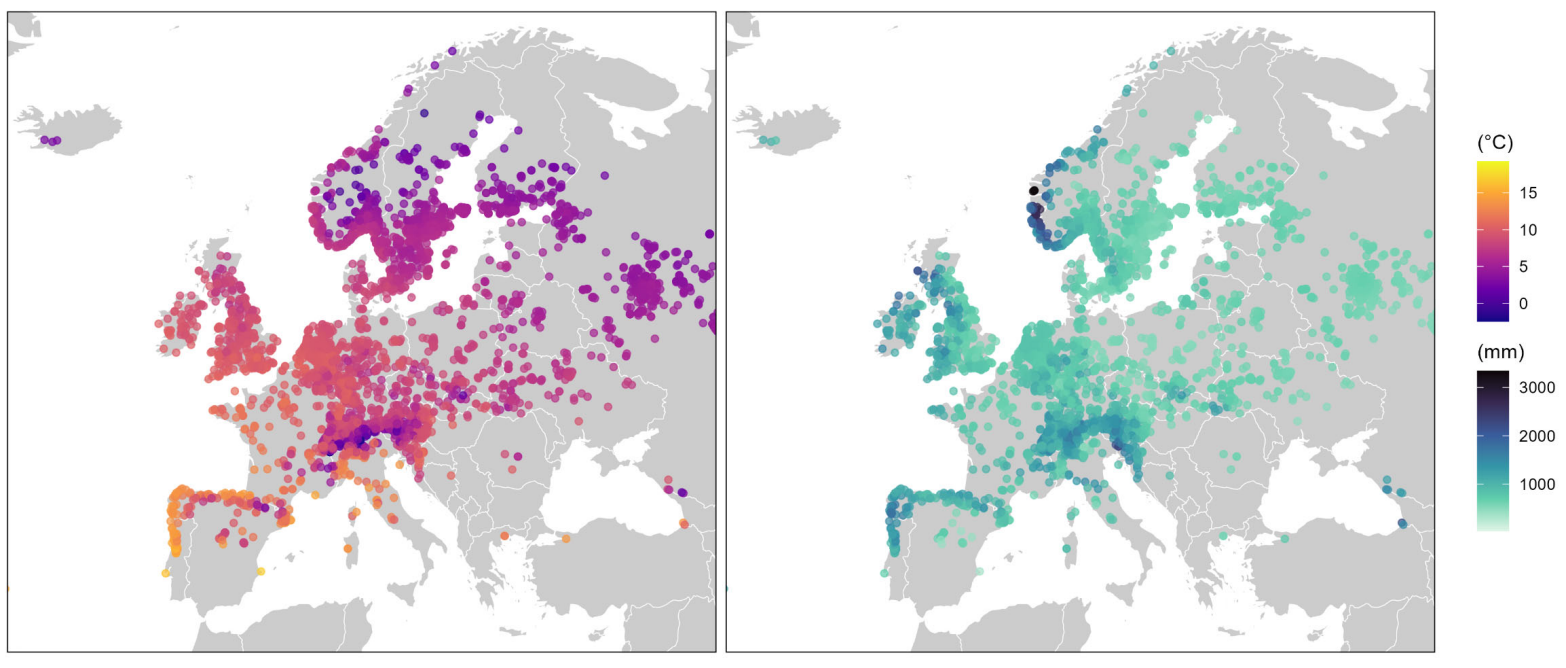
Geographic distribution and climatic gradients of *Fragaria vesca* across Europe. (left) Mean annual temperature (°C) at occurrence sites, colours range from cold (dark purple) to warm (yellow). (right) Annual precipitation (mm); colours range from low (light turquoise) to high (dark navy). Each point represents a GBIF occurrence record. Climatic data were extracted from WorldClim v2.1 using the geodata and terra R packages.

*F. vesca* is native to temperate North America, Europe, and northern Asia, occupying habitats from shaded woodlands to subalpine zones, on soils ranging from calcareous to acidic, and even disturbed sites (Liston et al., 2014; Hilmarsson et al., 2017; Fernie, 2023). Its circumboreal distribution spans from 37°N to 70°N latitude, encompassing broad environmental gradients (Njuguna et al., 2013; Faehn et al., 2023) (**Figure 3**).

About flowering, there is a well-established negative correlation between latitude and flowering earliness, such that populations from higher latitudes tend to flower earlier than those from more equatorial regions. This pattern reflects adaptation to progressively shorter growing seasons and harsher climates (Heide and Sønsteby, 2007; Still et al., 2023).

### 3.3 Advantages as a model system for eco-genomic research

*F. vesca* combines a small diploid genome (2n=2x=14; ~221 Mb) fully sequenced for comparative genomic studies (Shulaev et al., 2011; Zhou et al., 2023), with self-compatibility for generating inbred lines and efficient outcrossing for QTL mapping and recombinant inbred lines (RILs) development (Sargent et al., 2006; Slovin et al., 2009). Its small size makes controlled environment and common garden experiments easier to be conducted (Hancock and Bringhurst, 1978; Sammarco et al., 2022), and efficient clonal propagation capability provides replicates for robust G×E analyses (Sultan, 2000). Moreover, *F. vesca* amenability to be transformed by *Agrobacterium* mediated protocols, make it useful for functional genomics research (Razavi et al., 2012; Pantazis et al., 2013; Schaart, 2014).

### 3.4 Applied relevance and broader significance

Beyond basic research, *F. vesca* has strong significant applied relevance. It is closely related to the octoploid strawberry *Fragaria × ananassa*, serving as a genetic reservoir, helping breeding programs of the cultivated strawberry, facilitating the development of marker-assisted selection (MAS) and genomic selection (GS) approaches (Denoyes et al., 2017; Liu et al., 2023). Thus, studies of metabolic adaptation in *F. vesca* not only improve our understanding of plant eco-evolutionary processes but also offer real benefits for sustainability, resilience, and quality of strawberries and other related wild plants. A detailed translational pipeline from *F. vesca* gene discovery to *F. × ananassa* breeding is provided in Section 5.3.2.

### 3.5 *Fragaria vesca* as a plasticity studies model

Although the full extent of plasticity in *F. vesca* is still under study, its broad ecological distribution suggests a high degree of phenotypic flexibility across multiple biological levels (Hancock and Bringhurst, 1978; De Kort et al., 2020; Still et al., 2023). Evidence indicates that plasticity operates through distinct but interconnected pathways: immediate metabolic adjustments, longer-term epigenetic modifications, and intergenerational plasticity.

Concerning metabolic adjustments, studies on cold adaptation show that a 10-day cold treatment in *F. vesca* changed its core metabolism, increasing soluble sugars, aspartic acid, and amines like putrescine, while proline, unlikely in many other species, did not increase. Instead, galactinol and raffinose levels significantly rose in leaves and roots, suggesting species specific metabolic plasticity (Rohloff et al., 2012). Comparative studies between cold-tolerant (*F. daltoniana*) and cold-sensitive (*F. vesca*) *Fragaria* species further demonstrated that differential accumulation of abscisic acid (ABA) and glucose signaling through the *ABF2* transcription factor gene underpin divergent cold stress responses. *F. daltoniana* exhibits robust ABA accumulation coupled with significant upregulation of ABF2, which enhances glucose signal transduction and results in high levels of glucose and fructose accumulation that effectively mitigate cellular damage under low temperatures. In contrast, *F. vesca*, despite ABA activation, shows downregulation of ABF2 expression under cold stress, leading to reduced glucose signal transduction and a more limited metabolic response. This differential regulation of the ABA-ABF2 pathway, combined with higher ABA accumulation in F. daltoniana, explains the species’ superior cold tolerance capacity (Shen et al., 2022).

About longer timescales adaptations, it has been described that genome-wide methylation changes occur under multiple stress conditions including heat, cold, drought, and salinity, resulting in the loss of methylation in centromeric/pericentromeric regions and differential methylation of promoters linked to stress-responsive transcription factors (López et al., 2022). Methylation profiles in wild European populations correlate with climatic origin and pass down through clonal generations, influencing expression of growth and stress-related genes (Sammarco et al., 2024). Moreover, reciprocal transplant experiments demonstrate that modifying methylation patterns changes plant performance under varying environmental conditions, indicating a direct epigenetic role in local adaptation (Gu et al., 2016; Sammarco et al., 2022). Given the tight integration between metabolism, epigenetics, and quick physiological changes, these traits in *F. vesca* may exhibit greater plasticity than many morphological traits, which are often limited by structural and developmental factors. Understanding how these specific plasticity mechanisms work could lead to ways to understand how plants can withstand climate change.

## 4 Metabolism as the axis of plasticity and adaptation

Phenotypic plasticity in response to diverse environmental scenarios is determined by molecular and biochemical changes. Metabolic network acts as the intermediary between genomic variation and the expressed final-term phenotype, acting as a dynamic bridge that integrates genetic information and environmental signals into adaptive responses (Sweetlove et al., 2008). This metabolic network operates through two interconnected but functionally distinct layers: primary metabolism, which encompasses the fundamental biochemical pathways essential for basic cellular functions; and secondary metabolism, which produces specialized compounds for defense, signaling, and ecological interactions (Fernie and Pichersky, 2015). While secondary metabolites often receive attention for their roles in stress tolerance and plant-environment interactions, it is the primary metabolic network which forms the foundational framework upon which all other metabolic processes depend. Variations in metabolite concentrations and pathway fluxes serve as an immediate mechanism through which genotypes perceive, interpret, and translate environmental signals into adaptive phenotypes (Obata and Fernie, 2012).

### 4.1 The multifaceted and evolving roles of primary metabolism

Primary metabolism comprises conserved pathways essential for survival, growth, development, and reproduction (Buchanan et al., 2015). Beyond their traditional roles in energy provision, carbon skeleton supply, and building-block functions, primary metabolites are increasingly recognized as crucial signaling molecules and regulatory hubs that actively coordinate environmental responses and developmental programs (Salam et al., 2023).

Soluble sugars exemplify this dual role. Their fundamental function in energy supply is well characterized, but their role as signaling molecules is often underestimated, even though they modulate fundamental pathways involved in photosynthesis, carbon partitioning, nitrogen assimilation, growth, defense, and senescence (Hanson and Smeekens, 2009; Li and Sheen, 2016). Glucose and sucrose are notable examples, interacting extensively with hormonal pathways and stress signaling networks and acting as metabolic sensors of the plant’s energy status (Wind et al., 2010; Lastdrager et al., 2014). In strawberry, glucose, fructose and sucrose accumulate substantially under drought stress, indicating that osmotic adjustment through sugar accumulation serves as a core plastic response to water limitation (Sun et al., 2015).

Amino acids constitute another functionally diverse metabolite group. Regarding stress response, proline is the most prominent example, accumulating to high levels under osmotic stress and acting as a compatible solute, antioxidant, and regulator of stress-responsive gene expression (Szabados and Savouré, 2010; Hayat et al., 2012; Forde and Roberts, 2014; Hildebrandt et al., 2015). Notably, *F. vesca* exhibits species-specific amino acid responses to cold stress that diverge from those observed in model organisms (see Section 3.5). Similarly, GABA (gamma-aminobutyric acid) acts as a metabolic signal linking carbon and nitrogen metabolism (Shelp et al., 2012; Ramesh et al., 2015). Other amino acids also play stress-related roles; for instance, asparagine and glutamine serve as major nitrogen transport compounds and regulate nitrogen-responsive gene expression (Gaufichon et al., 2010; Krapp et al., 2011).

Organic acids, particularly TCA cycle intermediates such as malate, citrate, and fumarate, also have important regulatory role. They influence pH regulation and ion transport, and act as metabolic signals coordinating nitrogen metabolism, stomatal function, and plant-microbe interactions (Fernie and Martinoia, 2009; Meyer et al., 2010; Finkemeier et al., 2013). Moreover, they serve as alternative respiratory substrates and osmoregulatory compounds under stress conditions (Igamberdiev and Eprintsev, 2016).In strawberry, organic acid metabolism displays substantial plasticity during fruit development and ripening, with citric and malic acid levels regulated by sugar content and developmental stage, as reflected in dynamic changes in acid composition correlated with transcriptional reprogramming of metabolic genes (Wang et al., 2025). Geographic studies of wild *F. vesca* populations reveal substantial variation in the glucose/fructose ratio and in citric and malic acid content across different climatic regions, with both location and growing year significantly metabolite profiles (Akagić et al., 2020).

These findings indicate that primary metabolism is not merely a passive network supplying resources on demand, but an active, dynamic system that integrates environment and the plant’s internal state, contributing directly to the regulation of gene expression and physiological adjustment.

### 4.2 Metabolic plasticity: dynamic reconfiguration of biochemical networks

Metabolic plasticity is the capacity of an organism to dynamically reconfigure its metabolic network in response to both internal developmental factors and a environmental stimuli (Sweetlove et al., 2008; Obata and Fernie, 2012; Seebacher and Beaman, 2022). Rapid adjustments occur via allosteric regulation and post‐translational modifications, while slower shifts involve transcriptional changes. These regulatory layers (transcriptional, post‐transcriptional, post‐translational, allosteric, and compartmentalization) operate together to enable both immediate and sustained metabolic responses (Kaiser and Huber, 2001; Huber and Hardin, 2004; Friso and Van Wijk, 2015).

Primary metabolic plasticity is clearly observed in central carbon metabolism, where flux through glycolysis, the TCA cycle, and the pentose phosphate pathway can be rapidly redirected based on cellular demands and environmental conditions (Plaxton and Podestá, 2006; Araújo et al., 2012). This redirection is mediated by multiple regulatory nodes: (i) post-translational modifications (PTMs) of key glycolytic enzymes, such as those controlling fructose-2,6-bisphosphate levels, which modulate glycolytic flux to enhance NADPH production and antioxidant capacity under stress conditions (Smyth et al., 1984; Rider et al., 2004); (ii) allosteric regulation by AMP/ATP ratios and redox status (NADH/NAD^+^), which directly sense the cell’s energetic state (Geigenberger et al., 2010); (iii) transcriptional control by key metabolic regulators, such as the *bZIP* family transcription factors, which upregulate genes encoding gluconeogenic enzymes and stress-responsive amino acid biosynthetic pathways during sugar depletion, as well as *MYB* family members, which suppress growth-promoting anabolic pathways while promoting catabolic and defensive pathways under stress (Roy, 2015; Wang et al., 2022a). Similarly, amino acid metabolism shows remarkable plasticity, with pathways for proline, GABA, and branched-chain amino acids being strongly upregulated under abiotic stress (Fait et al., 2008; Trovato et al., 2008). These regulatory nodes integrate information from multiple environmental and internal signals, enabling rapid yet flexible metabolic reprogramming that maintains both immediate survival and long-term fitness during environmental fluctuations (Sammarco et al., 2023) (Mao et al., 2022).

Metabolomics and fluxomics provide snapshots of these dynamic shifts, revealing how primary metabolic pathways are rerouted under stress (Fiehn, 2002; Schauer and Fernie, 2006; Schwender, 2008). ¹³C-labeling experiments have shed light how carbon flux through primary pathways shifts under drought, cold, and nutrient stress (Gauthier et al., 2010; Watanabe et al., 2013). In *F. vesca*, comparative omics reveal that red genotypes coordinate a developmental switch from proanthocyanidin to anthocyanin biosynthesis by downregulating early phenylpropanoid genes and activating anthocyanin-specific genes during fruit ripening. In contrast, white genotypes fail to execute this switch, maintaining high expression of proanthocyanidin and flavonoid pathway genes while anthocyanin genes remain silent. This regulation is plastic, with environmental conditions modulating the timing and magnitude of the shift (Härtl et al., 2017). Secondary metabolite composition in *F. vesca* fruits encompasses nearly 100 volatile organic compounds, with genotype-specific profiles showing marked differences, indicating that biosynthetic allocation toward specific volatile classes is under strong genetic and potentially environmental control (Atazhanova et al., 2025).

### 4.3 Primary metabolites as the biochemical basis of phenotypic plasticity

Changes in primary metabolites often reveal plastic adjustments in growth, development, stress tolerance, defense, and resource allocation.

Under drought or salinity conditions, several sugars, polyols, and amino acids accumulate to maintain cellular turgor and protect against oxidative damage (Bohnert and Jensen, 1996; Szabados and Savouré, 2010; Krasensky and Jonak, 2012). These metabolites also function as molecular chaperones, stabilizing proteins and membranes under stress conditions (Hare et al., 1998; Chen and Murata, 2011). Under resource limitation, sugar allocation shifts from shoots to roots, altering root:shoot ratios and enabling enhanced nutrient acquisition (Rolland et al., 2006; Smith and Stitt, 2007; Eveland and Jackson, 2012).

pH variations also set stressful conditions for plants, not only for their physiological consequences, but the difficulty of obtaining determined nutrients whose availability is tightly influenced by the acidity of the soil. Strategies to face this involves different primary metabolites like malate, whose accumulation acts as a molecular buffer minimizing acidity changes; or citrate and other organic acids, which can chelate toxic metal ions and facilitate nutrient acquisition (Lopez-Millan et al., 2000; Ryan et al., 2001). Under phosphorus deficiency, organic acid exudation from roots helps to solubilize phosphate from soil minerals, allowing its uptake (Vance et al., 2003; Lambers et al., 2006).

Finally, storage compound allocation represents another main plasticity mechanism for plants. Starch accumulation in leaves and roots, along with fructan storage in some species, helps to cope with carbon availability fluctuation, remobilizing them when photosynthetic assimilation is limited (Stitt and Krapp, 1999; Stitt and Zeeman, 2012). The flux between starch synthesis and sucrose export reflects real-time integration of source-sink relationships and environmental conditions.

In summary, changes in the primary metabolome are not just passive consequences of environmental change but are often active, regulated responses that form the mechanistic basis of phenotypic plasticity. Understanding how environmental factors modulate metabolic networks, and how these changes translate into ecologically significant phenotypes, is essential for a comprehensive understanding of plant adaptation strategies and resilience, especially in species like *F. vesca*, which thrives across a wide spectrum of environmental conditions.

## 5 Deciphering the genetic basis: genome-wide association studies of primary metabolic traits

The study of G×E interactions for phenotypic traits is based on its heritable base (as described in section 2.3), which means that genetic variation among individuals or populations is the raw material upon which natural selection acts. However, just demonstrating G×E or estimating heritability does not elucidate the precise molecular mechanisms behind them. A main objective in modern biology is to identify the specific genetic loci that orchestrate these differential responses to the environment. Genome-Wide Association Studies (GWAS) has emerged as a key approach in this task, offering unprecedented power to dissect the genetic architecture of complex traits in natural populations, leaning on the development of global sequencies platform, which offers increasingly reduced costs and high reliability.

### 5.1 GWAS in plant metabolomics

GWAS establishes associations between genetic variants and phenotypic traits by analyzing historical recombination across natural populations (Korte and Farlow, 2013; Visscher et al., 2017). This approach relies on linkage disequilibrium (LD), where a marker near a causal variant serves as its statistical proxy (Slatkin, 2008). Modern approaches combine accurate trait phenotyping with high-density genotyping, while corrections for population structure and relatedness are implemented by using linear mixed models (Yu et al., 2006; Price et al., 2010). Multi-locus methods such as FarmCPU (Liu et al., 2016b) and BLINK (Huang et al., 2019) enhance power for polygenic traits, while integrating GWAS signals with metabolic networks helps prioritize candidate genes and improve biological interpretation (Toubiana et al., 2013).

### 5.2 mGWAS of primary metabolites: studying the genetic control of biochemical variation

The application of GWAS to identify genetic loci controlling variation in metabolite levels (mGWAS) has emerged as a powerful strategy for linking genotype to biochemical phenotype (Chan et al., 2010; Illig et al., 2010; Fernie and Tohge, 2017).

As metabolite concentrations are inherently quantitative, they can be measured with high precision employing techniques like mass spectrometry (GC-MS, LC-MS) and nuclear magnetic resonance (NMR) spectroscopy (Marshall and Powers, 2017; Karjalainen et al., 2024), reliable methods that have enabled massive meta-analyses, integrating data from thousands of individuals to increase statistical power (Riedelsheimer et al., 2012b; Karjalainen et al., 2024). In addition, due to the intermediary role of metabolism and the expressed final-term phenotype (view section 4), metabolite levels are often more directly linked to the function of specific genes encoding enzymes, transporters, or regulatory proteins, which allows mGWAS to more easily point to candidate genes (Riedelsheimer et al., 2012b; Wen et al., 2015).

In plants, mGWAS was first applied in *Arabidopsis thaliana*, identifying loci conferring resistance to pathogens, controlling amino acid levels and revealing genes in biosynthetic pathways and regulatory networks (Aranzana et al., 2005; Chan et al., 2010; Toubiana et al., 2012). Subsequently, it was expanded to other model organisms such as maize, where mGWAS of central carbon metabolites identified multiple loci affecting starch metabolism and revealing genetic networks coordinating carbon partitioning (Riedelsheimer et al., 2012a; Wen et al., 2015). Similarly, studies in rice have mapped loci controlling organic acid levels and amino acid composition, providing insights into nitrogen use efficiency and stress tolerance (Chen et al., 2014; Matsuda et al., 2015).

These early studies established a consistent pattern: primary metabolites exhibit strong genetic control with moderate to high heritability, making them excellent targets for genetic dissection (Riedelsheimer et al., 2012a; Chen et al., 2014; Matsuda et al., 2015; Wen et al., 2015). This heritability is especially relevant given their central role in fundamental life processes. For instance, variation in proline levels identified through mGWAS directly explains drought tolerance differences at the whole-plant level in *Arabidopsis* (Kesari et al., 2012). Moreover, modern approaches combinate mGWAS results with Mendelian randomization to establish causal relationships between primary metabolite levels and fitness-related traits (Hartwig et al., 2017).

Beyond identifying individual loci, mGWAS is a useful tool for the reconstruction of metabolic network architecture. For example, loci affecting both substrate and product levels can confirm enzymatic functions in amino acid biosynthesis or TCA cycle regulation (Do et al., 2010; Toubiana et al., 2012). Finally, mGWAS can identify regulatory genes controlling entire primary metabolic modules, revealing master regulators of carbon-nitrogen balance or stress-responsive metabolism (Gibon et al., 2006; Nunes-Nesi et al., 2010).

### 5.3 Key findings, applications and relevance to *Fragaria vesca*

*F. vesca* is a powerful system for GWAS-based dissection of adaptive and metabolic traits. Its favorable biological characteristics (see Section 3) make it an ideal bridge between ecological/evolutionary research and applied crop improvement (Samad et al., 2017; Davik et al., 2021; Gaston et al., 2021).

However, applying mGWAS to wild *F. vesca* populations requires careful attention to perennial-specific complications: cryptic population structure arising from historical geographic isolation and local adaptation can generate spurious associations between SNPs and metabolite levels if not rigorously controlled (Gloss et al., 2022). Clonal propagation via stolons means that phenotypic replicates may not represent independent genetic individuals, overestimating effect sizes while breaking statistical assumptions of independent and identically distributed observations (Liu et al., 2016a). Moreover, developmental asynchrony related to photoperiodic dormancy means that metabolite phenotypes are inherently stage-dependent and environment-specific, complicating standardization across genetically diverse populations (Clauw et al., 2025). Nevertheless, wild *F. vesca* being a perennial specie simultaneously offer unique advantages for mGWAS: clonal genotypes can be phenotyped repeatedly across multiple seasons and environmental conditions, enabling direct estimation of genotype-specific reaction norms (G×E slopes) as quantitative traits themselves, permitting “reaction norm GWAS” to identify genes controlling the magnitude and direction of metabolic plasticity (Liu et al., 2016a; Clauw et al., 2025). Moreover, primary metabolite variation discovered in cultivated strawberry (*F. × ananassa*), including loci for sugar content, organic acid balance, and amino acid profiles, are readily testable in the diploid *F. vesca* background, accelerating trait validation and functional follow-up (Vallarino et al., 2019; Alarfaj et al., 2021).

#### 5.3.1 Comparative perspective: primary metabolite GWAS in rosaceae crops

Although *F. vesca* offers unique advantages as a diploid model, it is part of the Rosaceae family, where GWAS has already provided relevant insights into fruit quality, phenology, stress adaptation, and metabolite diversity. **Table 1** summarizes representative studies focusing on primary metabolites, their main traits, key findings, and potential relevance to *F. vesca* research.

**Table 1:** Representative genetic and genomic studies of primary metabolites in Rosaceae species.

Species	Reference	Traits/ phenotypes analyzed	Key findings	Relevance/ link to *Fragaria vesca*
Apple (*Malus domestica*)	Liao et al., 2021	Soluble sugar contents, organic acids	Identification of genes associated with fruit taste	Orthologous genes conserved in *F. vesca* useful for mGWAS
	Wang et al., 2022c	Fructose, sorbitol	SNP affecting fructose content	Sorbitol dehydrogenase and its regulators as candidates for *F. vesca* studies
	Kumar et al., 2022	Organic acids	GWAS identifies multiple loci for metabolite variation.	Malic acid loci applicable to *F. vesca*
	Li et al., 2022	Sorbitol	Comparative evolutionary analysis	Conserved sorbitol metabolism gene family evolutionary patterns applicable to *F. vesca*
	Kunihisa et al., 2024	Sorbitol	Defects in sorbitol metabolism or transport induces extracellular sorbitol accumulation	Sorbitol transporters as candidates for sugar GWAS in *F. vesca*
	Watts et al., 2023	Sugars, organic acids (multiple ripening traits)	Large-scale GWAS reveals regulator of multiple ripening-related traits	NAC transcription factor as regulatory candidate for primary metabolites
Japanese pear (*Pyrus pyrifolia*)	Nishio et al., 2021	Soluble sugars	SNPs associated with large effect on sucrose and glucose	Conserved candidate genes in Pyrinae genomes with high genomic selection accuracy
Peach (*Prunus persica*)	Wang et al., 2022b	Organic acids	SNPs associated with organic acid content	Tonoplast transporters as key candidates for organic acids in *F. vesca*
	Jiang et al., 2023	Organic acids	Co-expression network analysis identifying candidate genes for organic acid metabolism	Organic acid regulatory networks transferable to *F. vesca*
	Etienne et al., 2002	Sugars and organic acids	QTLs for sugars and organic acids	Fundamental framework for primary metabolite QTL mapping in Rosaceae
	Baccichet et al., 2021	Organic acids	Characterization of 201 accessions for fruit quality traits with focus on acidity and organic acids	Phenotypic diversity for association studies in *F. vesca*
Apricot (*Prunus armeniaca*)	Jiang et al., 2025	Sugars and acids	Stable QTLs related to sucrose, glucose and fructose	Conserved transporters applicable to *F. vesca*
Sweet cherry (*Prunus avium*)	Holušová et al., 2023	Fruit firmness, sugar content	High-resolution GWAS locates candidate regions for quality and maturity traits	Fruit quality candidate genes applicable for *F. vesca* studies
	Gracia et al., 2025	Sugar and acid content	Genetic and QTL analyses of sugar and acid content	First genetic characterization of primary metabolites in non-climacteric Prunus
*Rosa roxburghii*	Su et al., 2023	Sugars and organic acids	Transcriptome and metabolome identifying key genes	Conserved sugar and acid synthesis genes regulatory transcription factors
Red raspberry (*Rubus idaeus*)	Durán-Soria et al., 2021	Amino acids, sugars and derivatives, organic acids, polyamines	GC-TOF-MS metabolomic profiling identifying primary metabolites G×E interactions	Methodological framework for primary metabolite studies applicable diversity to *F. vesca*
Blackberry (*Rubus* spp.)	Zurn et al., 2020	Sugar content	Rosaceae family-level candidate gene approach to identify genes associated with sugar content syntenic regions	Conserved candidate genes for sugars applicable across Rosaceae
Cultivated strawberry (*Fragaria × ananassa*)	Liu et al., 2020a	Soluble sugars	Characterization of genetic variation in fruit sugars	Sugar transporters and regulatory genes applicable to *F. vesca*
	Muñoz et al., 2024	Sugar content (Brix), organic acids	Diverse strawberry GWAS population positive correlation between sugars and Brix values	Diverse population to validate loci in *F. vesca*
	Pott et al., 2020	Amino acids and derivatives, organic acids	QTL mapping	mQTL methodology and hotspot regions applicable to *F. vesca*
	Lin et al., 2024	Citric acid, soluble protein	Regulation of citric acid and soluble protein content during ripening	Ripening regulatory genes affecting primary metabolites
Tomato (Solanum lycopersicum)	Vallarino et al., 2023	Amino acids, sugars, organic acids	Identification of genetic bases for amino acids, sugars and organic acids	mGWAS methodology and candidate genes applicable to *F. vesca*

These studies reveal several consistent patterns across Rosaceae species that should guide strategic research priorities in *F. vesca*. Sorbitol metabolism emerges as a Rosaceae-specific signature pathway: genes encoding sorbitol dehydrogenase (SDH) and sorbitol transporters appear repeatedly across apple studies (Li et al., 2022; Wang et al., 2022c; Kunihisa et al., 2024), reflecting the importance of sorbitol in Rosaceae. Given that *F. vesca* shares this evolutionary heritage, SDH orthologs and their transcriptional regulators represent high-confidence candidates for mGWAS targeting sugar allocation and stress tolerance. Another example implies organic acid regulation through tonoplast transporters; studies in peach (Wang et al., 2022b), apple (Kumar et al., 2022), and apricot (Jiang et al., 2025) identify vacuolar membrane transporters as major determinants of malic and citric acid accumulation, extremely valuable knowledge applicable to *F. vesca*, where fruit acidity is a key quality trait. Sugar transporter gene families show conserved associations with fructose:glucose ratios across apple, pear, strawberry, and blackberry (Liu et al., 2020a; Zurn et al., 2020; Liao et al., 2021; Nishio et al., 2021), suggesting that allelic variation at these loci underlies the substantial sugar composition diversity observed in wild *F. vesca* populations. Finally, NAC transcription factors emerge as master regulators coordinating primary metabolite shifts during fruit ripening (Watts et al., 2023), indicating that regulatory genes should be prioritized in *F. vesca* mGWAS designs targeting developmental plasticity. Altogether, these conserved pathways define a tractable target list for *F. vesca* research.

#### 5.3.2 Translational impact and broader significance for *Fragaria vesca* research

The identification of causal genes controlling primary metabolite variation through GWAS in *F. vesca* has key implications for cultivated strawberry improvement. Despite ploidy differences, knowledge from the diploid progenitor is invaluable for understanding fundamental metabolic processes and bridging genotype to phenotype in octoploid contexts. A practical translational pipeline operates as follows: first, mGWAS in *F. vesca* identifies candidate genes underlying metabolic traits. These loci are then used to search for orthologs and homeologs in *F. × ananassa* using the available reference genomes (Edger et al., 2019; Jung et al., 2019). Next, allelic variation at these loci is then characterized in breeding populations of the octoploid strawberry through targeted resequencing or SNP genotyping. Functional validation can be conducted in *F. vesca* and/or *F. × ananassa* using CRISPR/Cas9-mediated knockouts or RNAi-mediated silencing experiments to confirm causal relationships. Once validated, the most favorable variants are incorporated into MAS and GS models in cultivated strawberry, prioritizing alleles that enhance nutritional traits, stress resilience, and metabolic efficiency (Davis et al., 2006; Folta and Davis, 2006; Sargent et al., 2007). Following this generic pipeline, *F. vesca* has served as genetic reservoir for breeding traits like pathogen resistance, abiotic stress tolerance, and fruit quality (Slovin et al., 2009; Eikemo et al., 2010; Urrutia et al., 2017; Fernie, 2023).

Beyond crop enhancement, *F. vesca* serves as a model for understanding how primary metabolism underlies perennial plant adaptation, illuminating genetic trade-offs between growth, reproduction, and survival that are crucial for conservation of wild germplasm (Hancock and Bringhurst, 1978). Genes and primary metabolic pathways identified in *F. vesca* provide comparative insights into metabolic evolution across Rosaceae, leveraging synteny and conserved metabolic functions (Soundararajan et al., 2019).

#### 5.3.3 Translational impact and broader significance for *Fragaria vesca* research

mGWAS in *F. × ananassa* have mapped key primary metabolic genes controlling sugar content, organic acid balance, and amino acid profiles (Vallarino et al., 2019; Alarfaj et al., 2021; Wang et al., 2024). Loci controlling sucrose accumulation, citric acid content, and amino acid composition have been identified through large-scale metabolite QTL studies (Zorrilla-Fontanesi et al., 2012; Cockerton et al., 2018). Mapping these primary metabolite loci onto the *F. vesca* genome via synteny allows targeted resequencing of orthologs in wild populations to uncover novel allelic diversity affecting central carbon and nitrogen metabolism.

Once candidate variants are identified, *F. vesca*’s amenability to CRISPR/Cas editing, short generation time, and clonal propagation facilitate rapid functional validation of primary metabolic genes (Martín-Pizarro et al., 2019). Gene knockouts and allele swaps can confirm the roles of sugar transporters, amino acid biosynthetic enzymes, and organic acid regulators in metabolic plasticity and stress tolerance.

This reverse-translational pipeline specifically benefits primary metabolism research: cultivated strawberry discoveries of sugar metabolism and organic acid regulation generate hypotheses that are tested in *F. vesca*, and validated primary metabolite alleles can be reintroduced into breeding programs targeting improved nutritional content, metabolic efficiency, and stress resilience in both diploid and octoploid strawberries.

## 6 Future perspectives and conclusions

The study of plant adaptation, exemplified by *F. vesca*, is at an exciting turning point, driven by technologies that connect genetics, metabolism and ecology with greater resolution than bulk assays ever allowed. Beyond bulk tissue analyses that have obscured cell-type-specific responses, emerging technologies now offer unprecedented resolution to address fundamental questions about metabolic adaptation. Spatial metabolomics using advanced mass spectrometry imaging (MALDI, DESI-MSI) enables *in situ* visualization of metabolite distributions, providing insights into how specialized metabolite localization contributes to stress responses and ecological interactions (Boughton et al., 2016). In parallel, single-cell techniques are beginning to unravel cellular heterogeneity in plant tissues. For instance, single-cell transcriptomic atlases in *Arabidopsis* have already reconstructed developmental trajectories and stress responses (Denyer et al., 2019; Shahan et al., 2022), and even resolved phloem lineages at cellular resolution (Roszak et al., 2021). Applying these approaches to *F. vesca* will be crucial for deciphering how adaptive plasticity is orchestrated across multiple biological scales, from subcellular metabolite compartmentalization to whole-organism responses to environmental change.

To fully leverage *F. vesca* as a model for adaptation, research must move beyond identifying individual components to understanding system dynamics. Multi-omics integration offers the potential to address plasticity by mapping the flow of information from genetic variation (genome), through regulatory networks (transcriptome), to functional output (metabolome). By integrating enzyme kinetic parameters, transcriptomic regulation, and metabolic network information derived from mGWAS, researchers can build dynamic flux balance models that simulate metabolic rearrangement under stress conditions, which could predict: (i) whether populations can maintain essential carbon-nitrogen balance under different conditions; (ii) which genotypes exhibit sufficient metabolic plasticity to cope with extreme conditions; and (iii) where genetic trade-offs limit fitness, therefore identifying potential bottlenecks to climate adaptation. *F. vesca*, with its extensive natural genetic diversity and experimental tractability, serves as an ideal model for validating these predictive models before their application to more complex polyploid crops.

These integrative technological advances enable us to address several key questions that remain at the forefront of plant eco-genomics. First, which genetic networks drive metabolic adaptation beyond individual loci? While mGWAS has identified many casual variants, understanding how gene regulatory networks coordinate metabolic pathway responses under combined or sequential stresses remains elusive (Weiszmann et al., 2023). Second, how is plastic metabolism regulated in space and time? spatial metabolomics can reveal how allocation of defensive compounds or storage molecules shifts within organs and across developmental cycles, providing dynamic snapshots of metabolic reprogramming (Li et al., 2025). Third, what are the costs and limits of plasticity? Linking metabolic changes to fitness costs under different environmental scenarios will clarify the energetic and resource trade-offs that constrain adaptive potential (Murren et al., 2015). Finally, which metabolites function as integrative signals? Identifying key metabolites that act as integrative signals, analogous to trehalose-6-phosphate as sugar-status reporter, will clarify how environmental information is transduced into phenotypic responses (Figueroa and Lunn, 2016).

These advances create immediate opportunities for practical crop improvement, as metabolomics-assisted breeding can enhance prediction accuracy when integrated with genomic models. In major crops, metabolites have improved prediction of complex traits and heterozygosity (Riedelsheimer et al., 2012a; Xu et al., 2017). In strawberry, metabolic profiles can provide biomarkers for flavor, firmness, or stress resilience that accelerate selection when combined with SNP data. Furthermore, MAS benefits from octoploid discoveries that yield immediately deployable markers, with *F. vesca* serving to validate their function. For instance, breeders have employed FaFAD1 knowledge to improve strawberry aroma (Oh et al., 2021) and develop PCR-based diagnostics for *FaOMT* allelic variations affecting flavor notes (Zorrilla-Fontanesi et al., 2012). For polygenic traits such as yield, Brix, or firmness, genomic selection has proven extremely valuable in strawberry through multi-year and multi-environment training (Gezan et al., 2017; Osorio et al., 2021). Strategic integration of metabolite features as auxiliary predictors represents a logical next step towards multi-omic selection.

## 7 Conclusions

This review has highlighted the central, integrative role of metabolism as the biochemical nexus between genotype and adaptive phenotypes. We have established that phenotypic plasticity and local genetic adaptation operate in synergy, with metabolic networks providing the scenario where this interplay is realized. Changes in metabolite profiles represent the frontline of plastic responses to environmental change, while heritable genetic variations, identifiable through mGWAS, shape the capacity and regulatory efficiency of these pathways. *F. vesca* stands out as an exemplary eco-genomic model for dissecting how genetic variation cascades through the metabolome to drive ecological resilience. A comprehensive understanding of this multi-layered interaction is fundamental to evolutionary biology and provides a roadmap for sustainable improvement of agronomically important crops.
